# The Dental Protective Association: What It Has Done for Dentists, and Its Future Work in Their Behalf

**Published:** 1892-05

**Authors:** Louis Jack


### International Dental Journal.

Vol. XIII. May, 1892. No. 5.

### Original Communications.[1]

### THE DENTAL PROTECTIVE ASSOCIATION: WHAT IT HAS DONE FOR DENTISTS, AND ITS FUTURE WORK IN THEIR BEHALF.[2]

BY LOUIS JACK, D.D.S.

To enable the dental profession to understand the causes which led to the formation of the Dental Protective Association, it is necessary first to make an inquiry into the character and purposes of the International Tooth-Crown Company.

The first steps leading to the creation of said company are hidden in the brains of its originators, but the leading and active persons connected with it had occupied prominent and unenviable relations with the Goodyear Dental Vulcanite Company, and had learned by experience gained during its activity that in the field of dentistry was an opportunity to reap a rich harvest. They had found, by the apparent impunity with which they had harried the dentists of the country by the unfair means then pursued, how easy they might again lay the dental profession under tribute. They had also experienced the value of making threats to enforce upon individuals the pains and penalties of disregarding any *prima-facie*

[1] The editor and publishers are not responsible for the views of authors of papers published in this department, nor for any claim to novelty, or otherwise, that may be made by them. No papers will be received for this department that have appeared in any other journal published in the country.

[2] Read before the New York Odontological Society, February 16, 1892.

rights they might secure by the purchase of valid or invalid patent-claims.

These parties became organized under and pursuant to the laws of the State of New York, and it does not appear that such corporation is any other than a paper affair, organized for the purpose of holding by assignments large numbers of letters-patent relating to applied dentistry, and having no other objects in view than the gathering of profits by assessments and levies made upon the dental profession throughout the United States, under the guise of licenses for the use of procedures, so defended and producing the appearance of danger and peril to such dentists by reason of the aggregation of these various letters-patent, and also by the use of threats and the dread excited by the previous action of the Dental Vulcanite Company. Thus, then, in this manner, it is easily perceived was formed another hydra-headed corporation, which had furbished up the old plans of work and was already employing the agencies its congener, the Dental Vulcanite Company, had used to crush opposition, and were already showing the appearance of an effort to resort to similar means of control of both sides of the expected litigation, to the violation of the rights of the public.

At this point, to enforce this subject, it is well to refer to pages 26 and 27 of the Brief for Appellee in the case before the Supreme Court of the United States of the International Tooth-Crown Company *vs.* Edward S. Gaylord and John S. Williams, which read :

"The complainant corporation being in nowise a producer, or itself practising or employing the alleged inventions of its numerous patents, we assert with confidence that it is pertinent to the merits of this case and the object and merits of this complainant, that reference be made to the dental profession and the past relation and position of its members to the patents of this and analogous companies in this country, for more than a quarter of a century.

"It is a fact well known to most of the learned justices of this court, that, beginning more than a quarter of a century back, the individual members of the dental profession have been subjected to harassment and litigation in every Federal circuit of the United States, by corporations organized for the especial purpose of purchasing and holding patents relating to the various branches of dentistry, and demanding tribute from each member of the dental profession, and bringing suit in the Federal courts to enforce apparent claims in case of refusal. The principal illustration of this fact, so notorious and prominent that it is believed that this court will take judicial notice thereof, as a fact, was the litigation of the Goodyear Dental Vulcanite Company, carried on by a New York corporation for more than fifteen years against thousands of individual dentists in all parts of the United States.

"During that time the dockets of the various Federal Circuit Courts were simply clogged with these 'vulcanite cases.' A generation of dentists were impoverished by litigation, and there are still thousands of judgments standing upon the dockets of the courts obtained against younger members of the dental profession, so burdensome as to costs and judgments as to completely bar, or greatly discourage, any attempt of their payment or removal.

"This condition of affairs appears all the more unfortunate for the dental profession, when we consider that as a profession there are few, if any, of great or even ordinary wealth, and there are many of great poverty, wholly unable as individuals to defend themselves in any court; and that had the reissued letters-patent in that litigation been subjected to the same rule of construction, comparison, and analysis, as applied to reissued patents since the Miller-Bridgeport Brass Company case, its invalidity would seem to have been a matter of certainty.

"In the case of Smith *vs.* Goodyear Dental Vulcanite Co., 93 U. S. 502, the three learned justices of this court, delivering the dissenting opinion, used the following language:

"'It is too common a case that associated companies, in order to maintain some valuable monopoly, look about to see what abandoned invention or rejected application or ineffective patent can be picked up, revamped, and carried through the Patent-Office, and by the aid of ingenious experts and skilful counsel, succeed in getting the desired protection.'

"We most respectfully submit that the above language is peculiarly effective and correct, as descriptive of the acts and asserted patent property of this complainant, so far as such property is shown to exist by this record."

It mattered little to the Crown Company whether their claims were defensible or not. It was of little consequence whether some of these claims or their modes of enforcing them were plainly illegal or not. They knew well that the individual dentist is peculiarly weak when standing alone. This experience they had acquired during their enforcement of the claims of the Vulcanite Company. This Crown Company, therefore, prepared to magnify their efforts by the procurement of patents for procedures long in use, and by the purchase of other inventions of which it is now well known they were aware some were illegal and invalid, and which have since been so declared. The company, as stated, has also, in the effort to establish their claims, endeavored to procure questionable litigation by having control of both sides in a manner similar to the methods the Vulcanite Company attempted and nearly succeeded in imposing on the courts. This arraignment is presented to make clear the interests, nature, and the practices of this company, which being in nowise a producer or having any relation to the business of dentistry, has taken advantage of a situation where they have appeared intrenched behind the law, and where those on whom they would enforce their claims are isolated persons, so circumstanced by poverty and the absence of means of defence as to be nearly powerless.

The means the Crown Company has attempted to pursue and had effected upon many individuals is exhibited in the obligations, pains, penalties, and requirements they have imposed in the language of their licenses, the extreme terms and threats being the measure of the inherent weakness which pertains to most of their letters-patent held by assignment.

This said Crown Company entered into formal suits against Edw. S. Gaylord and Cassius M. Richmond, on what is known as the Richmond Crown patents and the Low Bridge patent. The defence of suit against Gaylord was carried on by Gaylord himself, until a committee was appointed in New York, when, by agreement, both suits were merged into one, and said committee took charge of the defence.

This committee raised and expended over five thousand dollars, and carried the case successfully through the United States Circuit Court, gaining a decision in favor of Dr. Gaylord and annulling the patent on crowns.

The case was argued before their honors, Judges Wallace and Shipman, in the October term of 1886, and was decided February 22, 1887. The bill was dismissed as to both the patents on the Richmond Crown, and on the preparation of roots of teeth for crowns. But the patent known as the Low Bridge patent was declared valid, and, according to the construction of that court, all other forms of permanent bridge dentures were declared an infringement of the Low Bridge.

As soon as these decisions were made public, the Crown Company issued circulars and started their agents with a view of issuing licenses and collecting royalty as though nothing had happened; making it appear to the masses of the profession that the decision of the court above spoken of was an entire victory for them. And by their peculiar methods they were procuring licenses from all parts of the country.

The signing of these licenses compelled those signing them to always recognize the validity not only of the Low Bridge and Richmond Crown patents, but also of some thirty-odd other patents owned by the company, which were included in this license-contract. In the mean time the Crown Company had appealed that part of the record relating to the Richmond Crowns which had been decided against them in the lower court to the Supreme Court of the United States.

It soon became apparent that something must be done, so a conference of several of the well-known members of the profession in New York and other parts of the country was held in New York, to meet the counsellors, Mr. S. J. Gordon and Mr. J. K. Beach, and discuss the situation. At this meeting Drs. McKellops and Crouse were urged to take up the work and relieve the committee in New York. It was at this meeting that Dr. Crouse agreed to take the matter under advisement, and also pledged himself to do something; but urged that it should be done by a larger proportion of the profession, in order to distribute the financial tax among a larger number; and also, so that those who could give valuable testimony would more willingly do so than they would to a local organization formed for that purpose.

The dental profession is indebted to Dr. Crouse for his energetic and self-sacrificing action in so promptly and effectively organizing what is now known as the Dental Protective Association. This Association was formulated in a manner so simple and effective in its nature that its action is little liable to interference, with every preparation for active defence and safeguards for the security of its funds. The officers are a president, vice-president, treasurer, secretary, and board of three directors. The by-laws invest large powers in the board of directors to insure prompt and energetic action, and to enable them to meet every emergency and device which may be attempted to be imposed upon the public.

To be ready to make fight whenever opportunity offers, and to resist by astute action each encroachment upon the rights of the public, and which could only be effective where the power to act is concentrated.

The principal purpose, as declared in the duties of the board of directors, is "to take charge of the defence of members of the Association in any of the States and Territories when prosecuted for the infringement of patents, the validity of which has not been fully established; and, with the funds of the Association, to retain and pay one or more counsel of their own selection, and to defray all necessary and proper expense of any such litigation."

The second object is to bind the dental profession together for mutual protection, strength, and helpfulness. The procurement of associated effort was based on the payment of ten dollars, and the obligation to pay ten dollars more should it become necessary at any time so to do. In case the membership should be of sufficient numbers, this latter payment would probably never be required.

At the end of the first year the membership was six hundred and sixty, which became nearly doubled the following year, and now is between three and four thousand.

The incorporation was executed in Illinois, and it is capable, through its board, of suing and being sued. It will be seen before this presentation is completed that it is a power in the land and before the law, and that it may become a shield and buckler against any wrong attempted to be imposed upon its members and the public.

THE WORK OF THE ASSOCIATION.

At the time of the organization of the Protective Association the International Tooth-Crown Company had commenced suits against six dentists in Milwaukee, five or six in Baltimore, and about as many more scattered over the State of New York. The Association assumed control of all suits commenced by the Crown Company against members of the profession, except when the parties were licensees of the Crown Company. The defence in these cases was avoided, because it was necessary to be sure of the loyalty of the defendants. The Association's attorneys made service on the Crown Company, and asked the court at Milwaukee to set the time when the question should be argued, and to set and limit the time when the testimony should be made up. The court limited the time to twenty days. Before the expiration of that time the Crown Company dismissed all the suits and paid the costs. The same steps were taken at Baltimore, with the same results, they dismissing the suits and paying the costs. This result was a great point gained in the struggle of resistance. Their avoidance of suit was not only an indication of the presence of the force which was pitted against them; it was also a confession of their own inability to sustain a suit to be made up anew upon a complete line of defence, supported by the matured evidence the Association had gathered with which to resist the claims of the Crown Company.

From the time of these dismissals the Association commenced taking licensees, regardless of their license-contracts, except in the circuit comprising New York and Connecticut. It certainly is a great result that the Association has thus relieved the dentists of the other eight circuits, so that they are to-day as free from the Crown Company's actions as though all their patents had been declared invalid.

It is easy to perceive the contrast between this and the corresponding period in the history of the Dental Vulcanite Company, when, because of the absence of organization of the dental profession, said company, in their efforts to establish their questionable claim, harassed the country from end to end, neither city nor town escaping,—no game being too small for them. After the operations of the Association commenced, the Crown Company endeavored to intimidate its officers and those aiding them by threatening suit for damage, and actually commenced suit against Dr. Crouse, president of the Association, for sixty thousand dollars and six months' imprisonment as damages. The grounds on which they based this claim for damages were "that the dental profession were satisfied with their methods and way of doing business; that Dr. Crouse had organized the Dental Protective Association out of spite towards the Crown Company, and, furthermore, that, by so doing, he had damaged their business."

This was a queer business which was being damaged,—the enforcement of claims for the use of inventions many of which they well knew, as before stated, were illegal, but the upholding of which were necessary to the further objects they had in view.

During the course of these events, the Association made it difficult for the Crown Company to get new licensees, whereupon they commenced to retaliate upon some of their former licensees who had joined the Association, hoping to get the Association into court as aiders and abettors in the vitiation of contracts. This legal complication was carefully avoided.

In connection with the motion of Mr. Offield, to limit the Crown Company suits at Milwaukee, he filed a cross-bill setting forth the character of the business the Crown Company were pursuing, and the questionable nature of the circular letters, advertisements, etc. These were filed in court, and it was asked that the Crown Company be enjoined from issuing any such documents. While the judge ruled this action out as irrelevant to the question of the validity of the letters-patent, he suggested it be brought up as a separate motion. He stated that no corporation had the right to issue such documents, which were for the purpose of intimidating and misleading the public.

The decision of Judge Jenkins, of the Milwaukee Court, is founded in justice and the rights of men. Corporations, from the very nature of their form and the absence of individual responsibility, tend to the impairment of the rights of those in the relation of defendant, since they proceed coolly to the execution of measures which an ordinary complainant would hesitate to institute. If, in addition to the force of their numbers and capital, they were allowed unrestrained action by their agents and of their threatening documents, the intimidation plainly would be in the direction of debarring the defendant from the free support of his case.

To evolve a clear idea of the character of these documents, it is only necessary to consider the form of their license and to analyze some of their advertisements. The Crown Company resorted to the ruse of employing dentists to give clinics at conventions and also to furnish instruction to dentists at their offices. When these agents became aware that the instructed parties were employing the procedures, they would follow up with their methods of inducements and intimidations. If successful in capturing the victim, the following or similar advertisement would appear in the local papers:

"DENTAL NOTICE.—This is to certify that Dr. Albert Robinson, Office No. 65 Monroe Street, is our only licensee to make and furnish to patients in the City of Grand Rapids any TOOTH-CROWNS, or BRIDGE-WORK, so called, manufactured under the patents of THE INTERNATIONAL TOOTH-CROWN CO. Said patents are constructed to cover the most practical forms of artificial Dentures now commonly known as Crown- or Bridge-Work.

"All persons are hereby cautioned against obtaining any such artificial Dentures from ANY DENTIST NOT LICENSED, as none are authorized except by the written license of said Company.

"The full legal penalty will be promptly enforced against all *Dentists* as well as their PATIENTS, making unauthorized use of any such patented Denture.

"A reward will be paid by said Company to persons furnishing it with any cases of BRIDGE-WORK of one two or more Crowns or Bridges made by any dentist not licensed by this Company.

"INTERNATIONAL TOOTH CROWN CO.,
"By JACKSON W. ALWARD, GENERAL MANAGER.

"Dated NEW YORK, Feb. 16, 1889,
"56 WEST 33RD STREET."

This pernicious advertisement sounds strangely in the ears of all men since the decision of the Supreme Court, which declared that whatever of invention these crowns possessed was only such modifications of old and well-known processes as pertained to the ordinary ingenuity of the workman in his capacity, and also that they had several years before been clearly and indisputably dedicated to the public.

As is well known, the Protective Association joined in the appeal to the Supreme Court of the suit of the International Tooth-Crown Company *vs.* Edw. S. Gaylord and John S. Williams, with the result that the Crown Company was beaten upon the claims of the Richmond Crown. In view of the evidence, it is surprising that an astute attorney would have carried their case to the Supreme Court, but the situation of the Crown Company was desperate. It was necessary for them, as before intimated, to secure the validity of the Richmond Crown, for on this was based not only what could be gained by the preservation of the patents covering these, but that without this method of making and securing crowns the other patents upon bridge-work would be rendered precarious. This plainly appears to be so, for the reason that the essential feature of bridge-work consists in the attachments of the bridge. If this were deprived of value to them, the whole plan of prosecution of their purposes would be in fatal danger.

The older forms of dentures were first made essentially as bridge-work is formed to-day, except the mode of attachment, the pieces often not firmly resting upon the gums, and the teeth being attached to bars which spanned the space. It is evident it was necessary, through thick and thin, by means fair or unfair, to make themselves secure upon the claims for the attachments, and they relied, as before, upon their peculiar methods both in and out of court, and upon the weakness of dentists. The result of this decision of the Supreme Court is that the Richmond Crown, with its various modifications, is free to all to use. In giving credit to the sturdiness of the defendants of this case, the dental profession is also under great obligations to the Dental Protective Association for the part they took in the case, and for the able brief with which it was presented to the court, and particularly so with reference to the clear and pertinent retrospect of the facts bearing on the case.

This brings us down to the present time in the history of the litigation.

It is not difficult to appreciate what would be the condition of the dentists of this country at this time who have been using Richmond Crowns in their practices, and who also have been making bridge-dentures, if the Dental Protective Association had not been in existence. Only the vigorous action of the Protective Association has blocked off the Crown Company from putting a great number of dentists under injunction or tribute, in the same manner as the Dental Vulcanite Company had done. They would have pushed their *prima facie* rights without much hinderance, and the persecution would have been in line with the previous experience of thousands of dentists.

The result has been that the members of the dental profession who have supported the Protective Association have been far more thoroughly protected on the small sum they have paid, than if they had expended a thousand or more dollars in their individual defence.

The second object of the Association, that of binding the dental profession together, has had considerable exemplification. This will become more and more manifest as the number of members is increased. What has already been done in this direction opens up the benefits to be secured in the future, when the dental profession becomes so united as to present an unbroken compact against wrong of whatever character from which it may suffer.

When the Dental Vulcanite Company commenced its exactions under claims which would not now be allowed, and which would not then have been permitted if the attorneys who managed the case had possessed sufficient astuteness, the dental profession was entirely without organized power, and the attempt in this direction which was made by the American Association was stopped by the one who assumed charge of the defence, by the advice that there was "no need of organization for such a purpose." The fatal error of this is now only too apparent.

It must be conceded as a marked result of the efforts of the Dental Protective Association, that the profession, at last, shows capacity to become organized for a given purpose.

It is now more than ever necessary to concentrate the power of the whole mass for defence, not only against the further unfair attempts of the Crown Company, but upon other equally illegal letters-patent.

This brings us to a consideration of the future work of the Association. There remains abundance for the Protective Association to do. That which immediately lies before it is the defence against the claims of the Crown Company concerning the Low Bridge patent, and to pursue this case to the court of final resort if it become necessary; to defend any of the Association who may be prosecuted until after the claims of the Crown Company are established in the higher courts; and, also, to test, as occasion may require, the validity of any patents of which the claims are of doubtful legality. That which has been done in the past by the Association is a warrant for the singleness, the sagacity, and the energy with which its actions will be guided in the future.

To give the Association the power to act with full force, and to have the reserve strength of abundant capital, it is the necessity of the hour that every one who is not now enrolled in the list of members, and who has any interest in dentistry, should join with the others. In this union is strength, a power not only of money, but of that far-reaching force, the inspiration of the moral impetus which the aggregation of intelligent men give to any cause in which they are deeply interested. The appeal now should be to every one who is not of the Association to become a member. The expense is small, the security is great, and the satisfaction is continual. Self-interest and duty each lay their claim upon every man to enroll himself, and to influence others to do the same.

The individual man in matters of litigation is weak, because he is without the knowledge, the opportunity to secure sufficient evidence, or the means to institute an effective defence; and in the face of the Crown Company or similar corporations, he is liable to be overwhelmed by the peculiar methods they pursue. He is like a single honey-bee, which is defenceless against the attack of an enemy and is killed in the effort, but when he becomes part of a colony, he bids defiance to attack and becomes an overpowering antagonist. In the same manner the units of dentistry may be, by the union of numbers, strong for defence against wrong, and effective to secure the rights of the public.

